# Protein Interaction Networks Reveal Novel Autism Risk Genes within GWAS Statistical Noise

**DOI:** 10.1371/journal.pone.0112399

**Published:** 2014-11-19

**Authors:** Catarina Correia, Guiomar Oliveira, Astrid M. Vicente

**Affiliations:** 1 Departamento de Promoção da Saúde e Doenças não Transmissíveis, Instituto Nacional de Saúde Doutor Ricardo Jorge, 1649-016 Lisboa, Portugal; 2 Center for Biodiversity, Functional & Integrative Genomics, Faculty of Sciences, University of Lisbon, 1749-016 Lisboa, Portugal; 3 Instituto Gulbenkian de Ciência, 2780-156 Oeiras, Portugal; 4 Unidade Neurodesenvolvimento e Autismo, Centro de Desenvolvimento, Hospital Pediátrico (HP) do Centro Hospitalar e Universitário de Coimbra (CHUC), 3000-602 Coimbra, Portugal; 5 Centro de Investigação e Formação Clinica do HP-CHUC, 3000-602 Coimbra, Portugal; 6 Faculdade de Medicina da Universidade de Coimbra, 3000-548 Coimbra, Portugal; Nagoya University Graduate School of Medicine, Japan

## Abstract

Genome-wide association studies (GWAS) for Autism Spectrum Disorder (ASD) thus far met limited success in the identification of common risk variants, consistent with the notion that variants with small individual effects cannot be detected individually in single SNP analysis. To further capture disease risk gene information from ASD association studies, we applied a network-based strategy to the Autism Genome Project (AGP) and the Autism Genetics Resource Exchange GWAS datasets, combining family-based association data with Human Protein-Protein interaction (PPI) data. Our analysis showed that autism-associated proteins at higher than conventional levels of significance (*P*<0.1) directly interact more than random expectation and are involved in a limited number of interconnected biological processes, indicating that they are functionally related. The functionally coherent networks generated by this approach contain ASD-relevant disease biology, as demonstrated by an improved positive predictive value and sensitivity in retrieving known ASD candidate genes relative to the top associated genes from either GWAS, as well as a higher gene overlap between the two ASD datasets. Analysis of the intersection between the networks obtained from the two ASD GWAS and six unrelated disease datasets identified fourteen genes exclusively present in the ASD networks. These are mostly novel genes involved in abnormal nervous system phenotypes in animal models, and in fundamental biological processes previously implicated in ASD, such as axon guidance, cell adhesion or cytoskeleton organization. Overall, our results highlighted novel susceptibility genes previously hidden within GWAS statistical “noise” that warrant further analysis for causal variants.

## Introduction

Autism Spectrum Disorder (ASD) is a complex neurodevelopmental illness with significant clinical and genetic heterogeneity. Family and twin studies demonstrated that ASD is one of the most heritable neuropsychiatric disorders, but there is yet no consensus on the underlying genetic architecture [1], [2]: while single-gene disorders, metabolic disorders and Copy Number Variants (CNVs) account for approximately 30% of the etiology of ASD [1], [3]–[7], the contribution of common risk variants to the remaining heritability is still unclear. Thus far, each large genome-wide association study (GWAS) carried out for ASD highlighted a single, non-overlapping locus [8]–[11], which frequently was not replicated by subsequent independent replication studies [12].

Devlin et al. (2011) have recently predicted that common variants having an odds ratio of 1.5 or more are very unlikely to exist; few, if any, common variants with an impact on risk exceeding 1.2 may still await discovery, but require much larger sample sizes, while variants with modest impact may range from zero to many thousands [13]. The small effect of common risk variants for ASD represents a challenge for their individual detection using conventional single-marker association analysis, which likely allows many true *loci* to remain hidden within the GWAS statistical “noise”. Evidence from classical quantitative genetic analysis further suggests that most of the heritability missing in complex diseases is rather hidden below the threshold for genome-wide significant associations [14], [15].

New strategies are therefore needed to increase the power of GWAS analysis. The use of molecular networks, which is not limited by *a priori* sorting the genes into incompletely annotated predefined gene sets, is emerging as an appealing unbiased alternative to pathway analysis. Network-based approaches have been widely applied in the analysis of high-throughput expression data from a wide range of diseases [16] and have proven successful in the identification of subnetwork markers more reproducible and with a higher prediction performance than individual markers [17]. More recent studies incorporated protein networks into the analysis of genome-wide association data, using networks to search for interacting *loci* in human GWAS data [18], [19] or to identify genome wide-enriched pathways [20]–[24]. However, an unsupervised global network analysis of ASD GWAS data that includes all signals without arbitrary significance thresholds has not been performed, and may lead to the identification of many risk variants of small effect below the accepted threshold for statistical significance.

Based on the premise that disease-causing genes are likely to be functionally related, in the present study we applied a network-based approach to two ASD GWAS datasets, the AGP consortium GWAS and the GWAS carried out in the Autism Genetic Resource Exchange (AGRE) dataset [10]. For this purpose we integrated genome wide association data with Human Protein-Protein interaction data and examined topological network properties indicative of connectivity at various levels of association, confirming our hypothesis that genes associated to ASD at a “statistical noise” level are functionally connected beyond random expectation. We compared the enrichment in known ASD candidates of network genes versus top GWAS genes, and the overlap of network genes vs the overlap at gene or SNP level between the two ASD datasets. The network obtained was further tested for ASD specificity using networks derived from six unrelated diseases GWAS, and explored for biological processes associated with ASD.

## Materials and Methods

**Figure 1:**
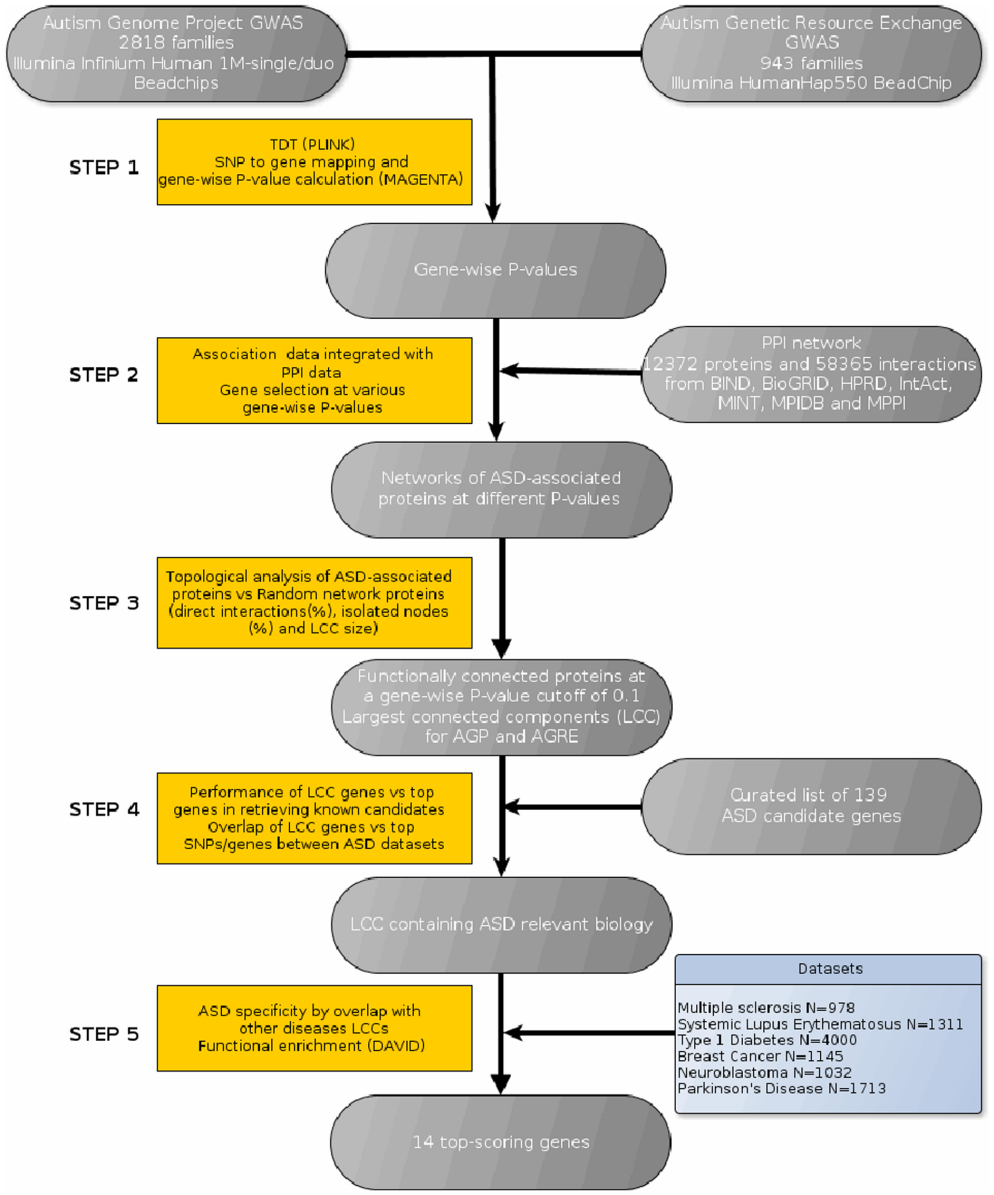
Workflow of the strategy for network definition, validation and identification of most relevant candidate genes.

A workflow of the strategy for network definition, validation and identification of the most relevant candidate genes is shown in Figure 1.

### Ethics statement

All the data used is previously published and publicly available. Written informed consent has been previously obtained from all families and procedures had approval from institutional review boards from all the institutions involved in recruitment and research, following national and international ethical and legal regulations and the principles of the Declaration of Helsinki.

### Datasets

The AGP dataset included 2818 trios consisting of autistic patients and both parents collected as part of the AGP Consortium. Patients were diagnosed and genotyped as previously reported [8]. Written informed consent was obtained from all families and procedures had approval from institutional review boards [8]. A total of 723 423 SNPs meeting the QC criteria [9], genotyped in 8491 individuals, were tested for association using the Transmissions Disequilibrium Test (TDT) implemented in PLINK v1.07 [25].

The GWAS replication dataset from the Autism Genetic Resource Exchange (AGRE) included 943 ASD families (4,444 subjects) from the AGRE cohort [10]. SNP genotyping data was obtained from AGRE [10]. Analysis in this study was limited to SNPs in common with the AGP GWAS and meeting the same QC criteria (425 587 SNPs).

Summary SNP association results were obtained from the database of Genotype and Phenotype (dbGAP) repository for 6 case-control GWAS for other pathologies, including Parkinson's Disease (PD) [26], Systemic Lupus Erythematosus (SLE) [27], Multiple Sclerosis (MS) [28], Type 1 Diabetes (T1D) [29], Breast Cancer (BC) [30] and Neuroblastoma (NB) [31] (Table S1). All individuals included were of European ancestry and the sample size was as similar as possible to the replication ASD dataset (AGRE).

### Integration of gene association data with Protein-Protein interaction data

Genotyped SNPs from the AGP and AGRE GWAS were assigned to specific genes if they were located within or up to 10 kb from the gene, using the GRCh37/hg19 genome build (Step 1). Each gene was assigned a gene score using MAGENTA (Meta-analysis Gene-set Enrichment of variant associations) [32], which allocates to each gene the most significant *P*-value among the TDT *P*-values of all individual SNPs mapped to that gene. MAGENTA then uses step-wise multivariate linear regression analysis to regress out of this *P*-value the confounding effects of gene size, number of SNPs per kilobase (kb), number of independent SNPs, number of recombination hotspots and the number of linkage disequilibrium units per kb.

Genes selected at various gene-wise *P*-value cutoffs (0.5<-LogP<5) were superimposed onto their corresponding protein on a large human protein-protein interaction (PPI) network, converting Entrez gene IDs to Uniprot IDs (release 2010_04) (Step 2). This PPI network, covering 12372 proteins and 58365 interactions, was previously built compacting data from six public PPI databases: BIND, BioGRID, HPRD, IntAct, MINT and MPPI [33]–[40].

### PPI network analysis

Topological properties from the resulting network were analyzed to select the gene-wise *P*-value for which corresponding proteins were functionally connected beyond random expectation, thus the lowest gene-wise *P*-value for which there is still relevant biological data in the GWAS that can be captured through network analysis (step 3). Three metrics indicative of this functional coherence were estimated for various association gene-wise *P*-value thresholds, for the two ASD datasets, and compared with those determined for 1000 equal size sets of randomly selected proteins from the human PPI network. The metrics evaluated were 1) the percentage of proteins directly interacting; 2) the percentage of isolated nodes, which represents the fraction of selected proteins with no interactions with any other selected protein; and 3) the size of the largest connected component (LCC), the largest group of selected proteins that are reachable from each other in the network. An empirical *P-*value was obtained computing the fraction of random samples where the value of the network metric is greater (or smaller in the case of isolates) than the observed one. Network analysis was performed using python module Network X.

### Performance against a candidate gene list and overlap between datasets

To evaluate the performance of the proteins included in the LCC in retrieving known ASD candidate genes, the precision and recall against a curated list of ASD candidate genes were calculated (step 4). This list was obtained from SFARIGene and includes 236 genes having at least minimal evidence of association with ASD (categories 1 to 4) or categorized as syndromic (https://gene.sfari.org/autdb/Welcome.do).

Precision (Positive Predictive value) is the proportion of known candidate genes among the selected genes, while recall (Sensitivity) is the proportion of known candidate genes retrieved by the selection. The precision and recall calculated for the genes encoding LCC proteins were compared to those determined using two other gene selection criteria: a) all genes selected at the same gene *P*-value cutoff used to derive LCC; b) the same number of top genes (ranked according to gene-wise *P*-values) as those included in the LCC.

Overlap between the AGP and AGRE datasets at SNP, gene and LCC levels was determined using the Jaccard index, defined as the size of the intersection divided by the size of the union of the datasets. For comparison purposes the size of each dataset LCC was used to select from each GWAS dataset an equal number of top SNPs (ranked by their TDT *P*-value) and top genes (ranked by their gene-wise *P*-value).

### Gene ranking and functional enrichment

To rank ASD-associated proteins included in the AGP LCC by ASD specificity and reproducibility, a prioritization system was created, assigning a score to each protein based on their presence in the LCC derived from the AGRE ASD replication dataset and from each of the six unrelated disease datasets (step 5). Each protein included in the AGP LCC had an initial score of 0.5. If the protein was present in the AGRE ASD dataset LCC, 0.5 was added to the initial protein score, whereas for each unrelated disease dataset LCC where the protein was present, one sixth of 0.5 was subtracted from the score. Therefore, protein scores vary between 0 and 1, with zero representing a protein present in the LCCs of the AGP dataset and the 6 unrelated diseases, while a score of 1 is attributed to a protein present only in the LCCs of both ASD datasets.

Functional enrichment was tested by DAVID (The Database for Annotation, Visualization and Integrated Discovery 2008_version6^th^; http://david.abcc.ncifcrf.gov) [41], [42], a publicly available bioinformatics tool that identifies functionally related groups of genes. Overrepresentation of mouse-mutant phenotypes was evaluated using the web tool MamPhea [43]. The complete list of the genes in the PPI network was used as background and *P*-values were corrected by the Benjamini correction. Top-scoring genes were further investigated using NextBio platform (Cupertino, CA, USA), a curated and correlated repository of experimental data derived from an extensive set of public resources (eg. ArrayExpress and GEO) [44]. Protein-protein networks were visualized in Cytoscape [45].

## Results

### Genes associated to ASD at *P*<0.1 are functionally related

Transmission Disequilibrium Tests were initially carried out in parallel for the AGP and AGRE datasets to identify small effect risk variants. In the sample of 2818 AGP families, single SNP transmission disequilibrium tests of the 723423 SNPs meeting the QC criteria showed no SNPs reaching the threshold for genome-wide significance. Two SNPs showed association signals at *P*<1×10^−6^ and very few exceeded *P*<1×10^−5^. In the AGRE dataset, after a similar quality control protocol and using only SNPs common to both datasets, three SNPs located in regions with no overlap with the AGP top findings showed association at *P*<1×10^−6^. Given the dearth of meaningful results from these two GWAS efforts, we proceeded with a network analysis strategy.

**Figure 2:**
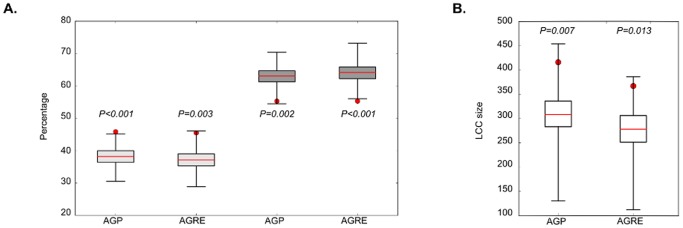
Network properties of proteins selected at gene-wise *P*<0.1 in each ASD. **a**) Comparison of percentage of direct interactions and isolated nodes between proteins selected at gene-wise *P*<0.1 in each GWAS dataset (red circles) vs 1000 random samples of network proteins (represented by light gray and dark gray box plots, for direct interactions and isolated nodes, respectively). The bottom and top of the box represent the 25th and 75th percentile and the extremity of the whiskers the maximum and minimum of the random samples data. **b**) Same comparison for the largest connected component (LCC) size.

The first step involved calculating gene-wise association *P*-values corrected for gene size and linkage disequilibrium, taking into account only the SNPs mapping within 10kb from each gene (403360 SNPs), followed by the integration of GWAS data onto protein interaction data. Then, we determined the lowest gene-wise *P-*value threshold for which genes encoding the network proteins were functionally related, inferred by their proximity in the network. Statistical noise is expected to have random connections in the network, while disease proteins are more likely to establish direct interactions between them and more rarely be isolated in the network, translating into a larger group of proteins that are all interconnected. For both ASD datasets, proteins encoded by genes selected at a gene-wise -Log_10_ *P* cutoff between 0.5 and 1.5 were found to establish significantly more direct interactions than equal sized sets of randomly selected proteins (Empirical *P* values 0.001<*P*<0.043), with the significance maintained up to -Log_10_ *P* = −2.0 in the case of AGRE dataset (Figure S1, Figure 2A). The number of isolated nodes was found to be significantly smaller in sets of ASD-associated proteins at the same range of gene-wise -Log_10_ *P*-values than in random sets (Empirical *P* values 0.001<*P*<0.038), again with significant differences maintained for lower gene-wise *P*-values in the AGRE dataset (Figure S1, Figure 2A). When compared to the same number of random proteins from the network, proteins encoded by genes selected at a gene-wise -Log_10_ *P*<1 from either ASD dataset are interconnected in a significantly larger LCC (Empirical P values 0.001<*P*<0.007) (Figure S1, Figure 2B). The large size of the largest connected components, 416 and 367 proteins for the AGP and AGRE datasets, respectively, indicates the existence of several small effect risk genes reinforcing the high genetic heterogeneity in ASD.

Based on the lowest gene-wise *P*-value for which the percentage of direct interactions was significantly higher, the percentage of isolated nodes significantly smaller and the size of the LCC significantly larger than random expectation (Figure 2A and B), we established gene-wise -Log_10_ *P* = 1 as the cutoff value to infer functional coherence from the two ASD datasets.

The overall results indicate that, as hypothesized, genes associated with ASD at the range of GWAS statistical noise encode proteins that are functionally related and preferentially directly interact, confirming our expectation that there is indeed unexplored relevant biology at this statistical level.

### Functionally coherent sub networks associated with ASD contain relevant ASD biology

To test whether the identified groups of functionally connected proteins captured by the largest connected components indeed contain ASD-relevant biology, we compared the performance of the genes selected through the LCC against a list of known candidates, [5] with the performance of all genes selected from the GWAS at the same gene-wise *P*-value cutoff or the performance of a number of GWAS top genes equal to the number of genes encoding LCC proteins. Genes implicated in ASD are largely unknown, thus low precision values are expectable given the incompleteness and noise in the available knowledge in the field.

Table 1 shows that, for both datasets, genes encoding proteins included in the LCC presented a 2 to 2.5 fold higher precision against the list of known genes than all the GWAS genes selected at the same statistical level cutoff. In other words, genes included in the LCC, and thus encoding functionally related proteins, are enriched in known candidates compared with the set of genes selected from the GWAS at the same statistical level, demonstrating that our filtering approach of association results based on PPIs more specifically captures ASD-relevant genes. A 1.3 to 3.3 fold increase is observed when comparing LCC genes with the same number of GWAS top genes, showing that a protein interaction-based selection was more accurate than selecting only the most strongly associated genes.

**Table 1:** Precision and recall were consistently higher for LCC genes relative to top GWAS genes or genes selected at *P*<0.1.

	Precision (%)		Recall (%)	
Gene subset	AGP dataset	AGRE dataset	AGP dataset	AGRE dataset
**LCC genes**	2.16	2.74	3.81	4.24
**GWAS Top genes**	1.68	0.82	1.27	2.97
**Genes selected at ** ***P*** **<0.1**	0.96	1,11	8.47	9.43

Precision and Recall (Percentage), by ASD dataset, of three sets of genes (genes selected at a gene wise *P*-value cutoff of 0.1, genes included in the LCC and the same number of GWAS top genes) against a list of known disease candidates.

Concerning the proportion of known genes that are retrieved by our selection, or recall, LCC encoding genes had a lower recall compared with all genes selected at the same cutoff, as expected since LCC genes are a subset of this selection (Table 1). However, compared with the top-gene selection, the 1.4 to 3 fold increase in the recall achieved by LCC encoding genes, indicates that additional relevant low effect genes are being captured. Further inspection of the known genes present in the top gene set and the LCC encoding genes confirmed that LCCs capture not only larger effect genes overlapping with top genes, such as *MET* (Uniprot P08581)(in AGP dataset), but additionally capture low effect genes, such as *TSC2* (Uniprot P49815), which single gene association analysis alone does not have the power to detect.

One of the major problems in ASD GWAS and GWAS in general is the low reproducibility of results between different datasets. Indeed, we found only one SNP (rs11837890 in *TBK1* gene) and 10 genes in common between the two datasets, when comparing the same number of SNPs or genes (ranked by *P*-values) than genes included in the LCCs from each dataset. Remarkably, we observed a 25 and 2.5-fold increase in the overlap between the two ASD datasets (AGP and AGRE) at PPI network level when compared to SNP or gene level, respectively (Figure S2).

Taken together, these results showed that our selection of functionally connected genes based on the largest connected component is an effective approach to capture ASD-relevant disease candidate genes, which might escape detection in an analysis based only on association evidence, even at gene-level.

### Functionally connected genes in ASD suggest novel susceptibility genes

Given the observation that the largest connected component contains ASD-relevant proteins, we further explored this network for biological processes implicated in ASD (step 5). The largest connected components generated by genes selected at -Log_10_ *P*<1 from the AGP and AGRE datasets comprised 416 and 367 proteins, respectively. A first look into the biological processes represented in these networks, using functional enrichment analysis, revealed an enrichment in pathways related to regulation of apoptosis and cell cycle. Additionally, intersection of the protein network data with knockout mice phenotypes from the Mouse Genome Informatics Database, showed that these proteins are primarily involved in aberrant embryogenic and developmental processes and anomalous immune system phenotypes.

A closer inspection of these LCCs at the gene level showed that around 30 (7–8%) of the encoding genes were implicated in neuropsychiatric or neurodegenerative disorders (Table S2). More interestingly, 20 (5–6%) of the LCC encoding genes were found to carry *de novo* mutations in ASD described in at least one of the three whole exome sequencing studies recently published [4], [7], [46], with 3 genes overlapping between the two datasets (*CSDE1* (Uniprot O75534), *PGD* (Uniprot P52209), *TSC2*). In addition, 80 (∼19%) of the AGP LCC-encoding genes were deleted or duplicated by CNVs identified by the AGP whole genome analysis as potentially pathogenic (with less than 50% of length overlap with control datasets) (Table S2).

To further examine the specificity of the proteins in the AGP LCC for ASD, this network was compared with LCCs generated from six unrelated diseases GWAS (MS, SLE, T1D, BC, NB, PD). Based on the presence of each protein in the LCC of each unrelated disease and in the AGRE LCC, we derived a highly stringent ASD-specificity protein score, allowing the prioritization of encoding genes for follow-up. Low scoring proteins were not replicated in the AGRE dataset, and were present in one or more unrelated diseases, whereas the highest scoring proteins were present in both ASD LCCs, but in none of the LCCs generated from the unrelated diseases. This analysis revealed that the majority of proteins (∼63%) were present only in the AGP network, while 31% of the proteins were present in at least one additional non-ASD network, and thus were not specific. From the 25 proteins identified in both ASD networks, the majority (56%) was not present in any ASD-unrelated network and 28% were present in one of the ASD-unrelated networks.

**Figure 3:**
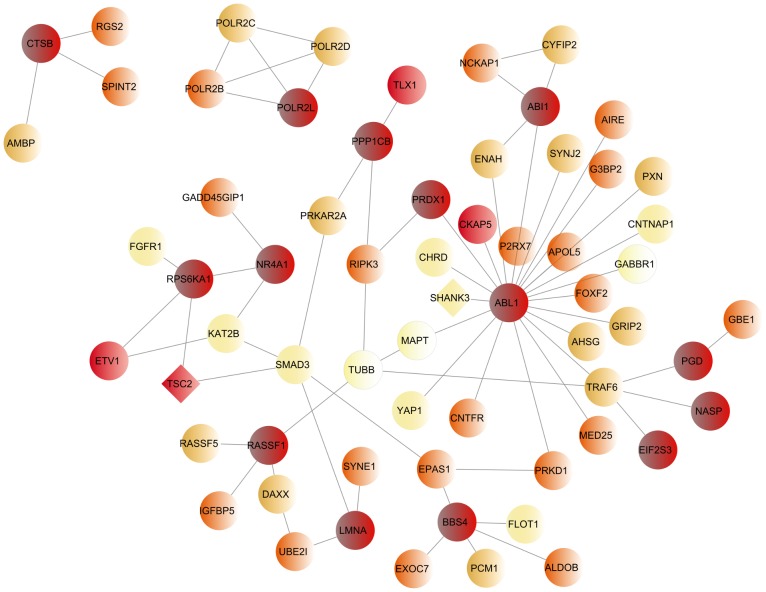
ASD top scoring gene network. This network illustrates the 14 top scoring genes included in the ASD LCC and their first neighbors. Nodes are colored based on a score reflecting their presence in the second ASD dataset and in the 6 unrelated diseases LCCs. A darker color represents a higher score, which means a higher specificity for ASD.

Using this gene scoring system, based on gene reproducibility and specificity for ASD, we built a network with the 14 top scoring genes and their first neighbors in the LCC network (Figure 3). The largest component of this network, although approximately 7 times smaller than the original LCCs, showed a similar overlap (∼5%) with genes reported to have *de novo* mutations in ASD (*PGD*, *SYNE1* (Uniprot Q8NF91), *TSC2*) and an increased overlap with known candidate genes (*SYNE1*, *TSC2* and *SHANK3* (Uniprot Q9BYB0)) and with genes contained in potentially relevant CNVs identified by the AGP analysis (∼26%). Enrichment in mouse phenotypes was also similar but, in addition, an enrichment in abnormal nervous system phenotype became significant, and in abnormal behavior/neurological phenotype borderline significant.

The genes encoding the 14 top scoring proteins were considered the best candidates for harboring common variants associated with ASD risk (Table 2). These genes are involved in various biological processes, such as NGF signaling, axon guidance, cell adhesion and migration, cytoskeleton regulation, apoptosis and DNA repair. A *de novo* mutation in the phosphogluconate dehydrogenase gene (*PGD*) has recently been reported in ASD [4], while potentially pathogenic CNVs deleting or duplicating the *ABL1* (Uniprot P00519), *RPS6KA1* (Uniprot Q15418) and *PPP1CB* (Uniprot P62140) genes were identified in ASD patients from the AGP study. A query of our genes in the NEXTBIO platform, a data mining framework that integrates and correlates global public datasets with several normal and disease phenotypes, revealed correlations of six genes with ASD. For instance, deletions within the *NASP* (Uniprot P49321) gene were identified in ASD patients from the Simons Simplex Collection (SSC) [47]. An altered expression of this gene, as well as of the *NR4A1* (Uniprot P22736), *ABI1* (Uniprot Q8IZP0), *BBS4* (Uniprot Q96RK4), *LMNA* (Uniprot P02545) and *ABL1* genes, was found in postmortem brain tissue [48] or lymphoblastoid cells [49] of ASD patients. Some of the 14 top-scoring genes, namely the *CTSB* (Uniprot P07858), *BBS4, LMNA* and *ABL1* genes, were associated with abnormal nervous system phenotypes in animal models. The most strongly associated genes to ASD, using the AGP data, were the peroxiredoxin 1 gene (*PRDX1* (Uniprot Q06830)) and cathepsin B gene (*CTSB*).

**Table 2:** Top scoring ASD network genes.

Gene (Uniprot ID)	Description	Location	Relevant biological processes	Gene-wise association *P*-value (MAGENTA)	Published studies in autism	Neurological and behavioral features in mouse models
*NASP(P49321)*	nuclearautoantigenicsperm protein (histone-binding)	1p34.1	blastocyst development, cellproliferation, cell cycle	1.470e^−02^	NEXTBIO: deletion inidiopathic females(Sakai et al, 2011), significantlydownregulated in brainsamples (Chow et al.2011)	NA
*PRDX1(Q06830)*	peroxiredoxin 1	1p34.1	redox regulation, cellproliferation	1.760e^−04^	-	NA
*RPS6KA1(Q15418)*	ribosomal proteinS6 kinase, 90kDa, polypeptide 1	1p36.11	protein kinase, synaptictransmission, axon guidance, long-term potentiation, toll-likeand NGF receptor signaling pathway	2.422e^−02^	-	NNP
*PGD(P52209)*	phosphogluconate dehydrogenase	1p36.22	cell redox regulation	7.851e^−02^	De novo mutation in autistic patient (O′Roak et al. 2012)	NNP
*LMNA(P02545)*	lamin A/C	1q22	regulation of cell migration, regulation of apoptotic process, spermatogenesis	8.226e^−02^	Nextbio:altered expression in Lymphoblastoid cells from males with autism (15q11–13 duplication) and brain samples (Chow et al. 2011; Nishimura et al. 2007)	abnormal axon morphology, abnormal myelination
*PPP1CB(P62140)*	protein phosphatase 1, catalytic subunit, beta isozyme	2p23	regulation of cell cycle, focal-adhesion, long-term potentiation, regulation of actin cytoskeleton	3.749e^−02^	-	NA
*RASSF1(Q9NS23)*	Ras association (RalGDS/AF-6) domain family member 1	3p21.3	Cell cycle, response to DNA damage stimulus, positive regulation of protein ubiquitination	1.949e^−02^	-	NA
*CTSB(P07858)*	cathepsin B	8p23.1	regulation of apoptotic process, cellular response to thyroid hormone stimulus	2.343e^−03^		Purkinje cell degeneration, abnormalneuron apoptosis (details)neuron degeneration
*ABL1(P00519)*	c-abl oncogene 1, non-receptor tyrosine kinase	9q34.1	axon guidance, regulation of cell adhesion, motility, cycle, actin cytoskeleton organization, response to DNA damage stimulus	9.560e^−02^	NextBio:altered expression in autistic brain samples (Chow et al. 2011)	abnormal cerebellum morphology, small cerebellum, abnormal cerebellum development, abnormal cerebellar foliation, ectopic Purkinje cell, abnormal cerebellar lobule formation, absent cerebellar lobules, abnormal neuron differentiation
*ABI1(Q8IZP0)*	Abl-interactor 1 previously known as spectrin SH3 domain binding protein 1	10p12.1	transmembrane receptor protein tyrosine kinase signaling pathway, negative regulation of cell proliferation	7.352e^−02^	NextBio:downregulation in autistic brain samples (Chow et al. 2011)	NA
*POLR2L(P62875)*	polymerase (RNA) II (DNA directed) polypeptide L, 7.6kDa	11p15.5	DNA repair, regulation of transcription	7.821e^−02^	-	NNP
*NR4A1*(P22736)	nuclear receptor subfamily 4, group A, member 1 also known as nerve Growth factor IB (NGFIB)	12q13.13	nuclear transcription factor, epidermal and fibroblast growth factor receptor signaling pathway, nerve growth factor receptor signaling pathway	4.783e^−02^	NextBio:downregulation in autistic brain samples (Chow et al. 2011)	NA
*BBS4(Q96RK4)*	Bardet-Biedl syndrome 4	15q22.3–q23	centrosome organization, microtubule cytoskeleton organization, neural tube closure, dendrite, striatum, hippocampus, cerebral cortex development	7.511e^−02^	Nextbio:altered expression in lymphoblasts and brain samples (Chow et al. 2011; Nishimura et al. 2007)	abnormal neural tubemorphology/development, thincerebral cortex, abnormal basal ganglion morphologyabnormal olfactory neuron morphology, small hippocampusenlarged lateral ventriclesenlarged third ventricle
*EIF2S3(P41091)*	eukaryotic translation initiation factor 2, subunit 3 gamma, 52kDa	Xp22.11	cellular protein metabolic process	7.322e^−02^	-	NNP

List of the 14 top scoring ASD network genes, present in both ASD networks and in none of the other disorders (ASD specificity score = 1), with information on gene-wise association *P*-value and biological processes relevant for ASD.

NNP - No neurological phenotypes|; NA - No mouse model available.

## Discussion

In this study we have conducted a network-based analysis of two ASD GWAS datasets, hypothesizing that small effect ASD risk variants hidden at the level of GWAS statistical noise can be discovered from networks of genes with related biological functions. Mapping of association data to a PPI network indeed revealed that, in both datasets, ASD-associated genes at *P*<0.1 encoded proteins that directly interact beyond random expectation, are more rarely found isolated in the network and are connected in significantly larger LCCs than expected by chance, suggesting a functional connection. These results support recent findings from the AGP consortium, showing that stronger association of allele scores with case status was generally achieved when those scores were based on markers associated at significance thresholds higher than 0.2 [8]. The International Schizophrenia GWAS consortium had similar results of optimal discrimination between cases and controls only after the inclusion of markers with *P*-values as high as 0.2, [14] using this allele scoring approach.

The relevance to ASD of these networks was further illustrated by their higher performance in retrieving known ASD candidates compared to top GWAS genes, and the increased similarity between the two ASD datasets, when compared to SNP or gene level overlap. Remarkably, the AGP and AGRE LCCs included 20 genes, respectively, in which *de novo* mutations have been described in whole-exome sequencing studies of nearly a thousand ASD patients [4], [7], [50]. A large overlap of our results with the published data of these sequencing studies was not expected, because the LCCs encoding genes are likely to harbor variants transmitted by unaffected parents, whereas these sequencing studies mainly focused, and reported only, *de novo* variants which do not explain the heritability of the disorder, but support recent observations that common and rare variants associated with ASD disturb common neuronal networks [51]. Moreover, around 20% of the AGP LCC encoding genes were deleted or duplicated by potentially pathogenic CNVs detected in the AGP whole genome CNV screening of 2446 ASD patients.

As an additional filter for meaningful ASD biology, we derived an ASD candidate gene prioritization system ranking the genes encoding proteins included in the AGP LCC for ASD reproducibility and specificity. The scoring system used was very stringent, in particular since some of the control disorders are neurological (Parkinson's, multiple sclerosis or neuroblastoma) and may share susceptibility genes and pathways with autism [52]–[56]. While we may have discarded relevant autism risk genes that are ubiquitous and common to these disorders, we believe that we enriched our list of genes in true positive results with a higher chance of experimental validation. In fact, the enrichment analysis performed with the top-scoring genes and their first neighbors showed a high content in mouse genes associated with nervous system or neurological phenotypes and a similar or higher overlap with candidate genes or genes reported with *de novo* mutations or potentially pathogenic CNVs in ASD.

This approach generated a list of 14 top-scoring genes, present in the two ASD networks and none of the other disorders, which were considered strong candidates to harbor common variants associated with ASD risk. These genes are mostly novel candidates for ASD, and are involved in nervous system pathways or other more fundamental biological processes which have been widely associated to ASD, such as ubiquitination [4], [9], [57], [58], cytoskeleton organization and regulation [5], [47], [59] and cell adhesion [10], [60]. For instance, the *CTSB, BBS4, LMNA* and *ABL1* genes have been associated with neurobiological phenotypes identified in an enrichment analysis of mouse neurobiological phenotypes from a list of 112 ASD candidate genes [61], with *CTSB* and *ABL1* associated with cerebellum morphological and development abnormalities. The AGP genome-wide analysis identified potentially pathogenic CNVs spanning *ABL1*, *RPS6KA1* and *PPP1CB*, whose relevance needs to be further established. Likewise, in the phosphogluconate dehydrogenase gene (PGD), a *de novo* mutation has recently been reported in a patient with ASD [4], although with an uncertain deleterious effect. This gene plays a critical role in protecting cells from oxidative stress [62] and, together with *PRDX1*, which also has an important antioxidant protective role in cells [63], [64] and shows the strongest association with ASD, supports emerging evidence for a role of oxidative stress in ASD pathophysiology [65], [66].

Thus far the use of protein networks to address common risk variants in ASD was limited to enrichment analysis of GWAS top hit genes in co-expressed or differentially expressed networks [51], [67]. In contrast, this study incorporated protein interaction data into GWAS analysis, without *a priori* assumptions of association thresholds. The present results have shown that autism-associated genes at higher than conventional levels of significance are functionally related, and were used to extract relevant disease biology and uncover small effect variants contributing to the disorder. The study highlighted a group of novel susceptibility genes relevant for CNS function with a high probability of bearing common variants associated with autism, which have been elusive thus far, and warranting further analysis for identification of causal variants.

## Supporting Information

Figure S1 **Network properties per gene-wise** ***P*** **-value for each ASD dataset.** For each –Log_10_ gene wise association *P*-value cutoff in the x-axis, the percentage of direct interactions (A) and isolated nodes (B) and the logarithm of the LCC size (C) were plotted for proteins encoded by disease-associated genes (red line) and for the mean of 1000 equal sized random samples of proteins (blue line). Dark grey areas represent the range between the 25th and 75th quartiles and light gray areas indicate the range between the minimum and maximum values of the random data. Empirical *P*-values are indicated for each gene wise association *P*-value comparison. Values are plotted until the –Log_10_ for which the percentage of direct interactions and isolated nodes reaches 0 and 100%, respectively.
(TIF)

Figure S2 **Overlap between the two ASD datasets at SNP, gene or network level.** Venn diagrams showing the overlap between the two ASD datasets (AGP and AGRE) at SNP, gene or network level.
(TIF)

Table S1 **GWAS datasets used in the analysis and genotyping platforms.**
(XLSX)

Table S2 **AGP LCC network genes.** List of the 416 genes included in the AGP LCC with information on gene-wise association *P*-value, specificity score for ASD and previous findings regarding implication in ASD and other neurological disorders.
(XLSX)
