# Supplementary figures and images for: Protein Interaction Networks Reveal Novel Autism Risk Genes within GWAS Statistical Noise
Source: PLoS One. 2014 Nov 19;9(11):e112399. doi: 10.1371/journal.pone.0112399 (PMC4237351; doi:10.1371/journal.pone.0112399)

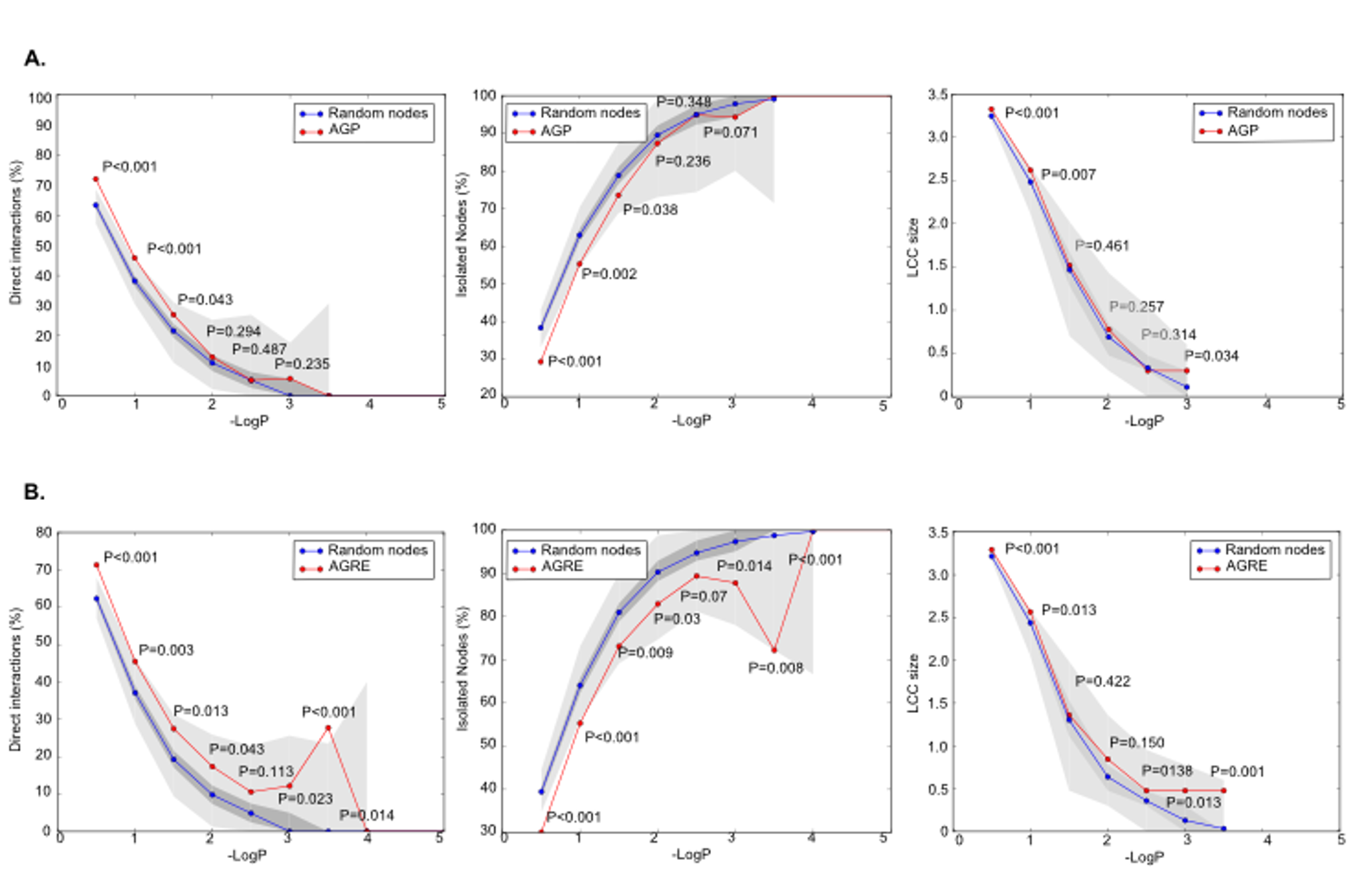

Supplement: Figure S1 — Network properties per gene-wise P -value for each ASD dataset. For each –Log10 gene wise association P-value cutoff in the x-axis, the percentage of direct interactions (A) and isolated nodes (B) and the logarithm of the LCC size (C) were plotted for proteins encoded by disease-associated genes (red line) and for the mean of 1000 equal sized random samples of proteins (blue line). Dark grey areas represent the range between the 25th and 75th quartiles and light gray areas indicate the range between the minimum and maximum values of the random data. Empirical P-values are indicated for each gene wise association P-value comparison. Values are plotted until the –Log10 for which the percentage of direct interactions and isolated nodes reaches 0 and 100%, respectively. (TIF) [file pone.0112399.s001.tif]
